# Suppressed Autoxidation, Enhanced Antioxidant Activity, and Improved Cytocompatibility of Epigallocatechin Gallate via Alginate Site-Specific Conjugation with Tunable Substitution Degree

**DOI:** 10.3390/ijms26178725

**Published:** 2025-09-07

**Authors:** Nunnarpas Yongvongsoontorn, Maho Kihara, Masaya Inada, Joo Eun Chung, Motoichi Kurisawa

**Affiliations:** Graduate School of Advanced Science and Technology, Japan Advanced Institute of Science and Technology, 1–1 Asahidai, Nomi 923–1292, Ishikawa, Japans2410010@jaist.ac.jp (M.I.); chungje@jaist.ac.jp (J.E.C.)

**Keywords:** epigallocatechin-3-gallate (EGCG), alginate, antioxidants, autoxidation, conjugation, natural polymers

## Abstract

Epigallocatechin-3-gallate (EGCG), a major polyphenol in green tea, exhibits strong antioxidant activity but suffers from poor stability due to rapid autoxidation under physiological conditions. In this study, we developed alginate–EGCG conjugates via a site-selective thiol-quinone addition reaction under mild aqueous conditions. The conjugation preserved EGCG’s flavanic structure while enabling tunable degrees of substitution (DS). We systematically evaluated the oxidative stability, antioxidant activity, and cytocompatibility of alginate–EGCG conjugates in comparison with free EGCG and a mixture of EGCG and alginate. Alginate–EGCG conjugates significantly suppressed EGCG autoxidation, reduced hydrogen peroxide generation, and improved cytocompatibility in human renal epithelial cells, especially at a low DS. Furthermore, alginate–EGCG conjugates retained or even enhanced superoxide anion radical scavenging activity, with higher DS conjugates exhibiting greater antioxidant effects. In addition, dynamic light scattering analysis revealed DS-dependent particle formation via self-assembly. These findings demonstrate that covalent conjugation with natural polymers is an effective strategy to improve oxidative stability and biological functionality of plant-derived polyphenols, offering a promising approach for developing advanced antioxidant materials for food, cosmetic, and biomedical applications.

## 1. Introduction

Epigallocatechin-3-gallate (EGCG), a predominant catechin in green tea, is widely studied for its strong antioxidant, anti-inflammatory, antimicrobial, anticancer, and neuroprotective effects [1,2,3]. These biological activities arise largely from EGCG’s polyphenolic structure, which includes multiple hydroxyl groups capable of scavenging reactive oxygen species (ROS) and stabilizing free radicals through hydrogen atom or electron transfer [4,5,6]. Owing to these properties, EGCG holds promise in diverse applications, including food preservation, cosmetic stabilization, and therapeutic interventions for oxidative stress-related problems [7,8,9,10]. Despite its beneficial properties, EGCG is highly unstable under physiological and environmental conditions. It rapidly undergoes autoxidation in aqueous environments, particularly at neutral to alkaline pH, or in the presence of oxygen, light, and metal ions [11]. This autoxidation leads to the formation of semiquinone radicals, dimers, and quinone derivatives, often accompanied by a loss of antioxidant function and the potential formation of cytotoxic byproducts [12,13]. These factors severely limit the shelf life, safety, and efficacy of EGCG in formulations of nutraceutical, therapeutic, antioxidant, or functional food additive applications [14]. Furthermore, EGCG is poorly soluble in water and sensitive to metal ions and enzymatic degradation, complicating its incorporation into stable formulations.

To address these challenges, polymer-based stabilization techniques have been increasingly explored. Especially, natural polymers offer the advantages of environmental sustainability and functional versatility. Natural polysaccharides such as alginate, chitosan, pectin, and cellulose derivatives offer excellent biocompatibility, biodegradability, and regulatory advantages (e.g., Generally Recognized As Safe (GRAS) status) [15,16,17]. These polymers are already employed across food, cosmetic, biomedical, and packaging applications as emulsifiers, encapsulants, film-formers, or structural scaffolds [18,19,20]. Among them, alginate, a linear anionic polysaccharide extracted from brown seaweed, is composed of β-D-mannuronic acid (M) and α-L-guluronic acid (G) units [20]. Its unique ability to form ionotropic gels in the presence of divalent cations, combined with abundant hydroxyl and carboxyl groups, makes it a valuable platform for conjugation or interaction with bioactive compounds [20,21,22]. Alginate is widely used in food coatings, wound dressings, biosensors, and cosmetic gels, and increasingly studied as a natural antioxidant carrier [23,24,25,26].

Non-covalent EGCG–polymer mixtures have shown modest improvements in solubility and short-term stability, but their weak and reversible interactions (e.g., hydrogen bonding) are prone to dissociation under stress [27,28]. In contrast, covalent conjugation offers longer-lasting stabilization by immobilizing EGCG within the polymer matrix and shielding its reactive groups from degradation [27,28,29,30]. However, many covalent strategies, such as carbodiimide-mediated amidation or esterification, target hydroxyl groups essential for EGCG’s antioxidant function, risking functional loss [31,32]. Thus, mild, site-selective reactions under aqueous conditions are essential to preserve activity during conjugation [29,33,34]. The stabilization mechanism of polymer–EGCG conjugates is multifaceted: steric hindrance by the polymer can physically protect EGCG, while electron delocalization may suppress ROS-mediated oxidation. Moreover, reduced molecular mobility in the polymer network may slow autoxidation pathways. In some cases, the polymer itself may contribute synergistically to antioxidant effects [35,36].

Despite growing interest in polyphenol–polymer hybrids, there remains a scarcity of studies on covalently conjugated alginate–EGCG systems. Existing work largely focuses on non-covalent complexes, with recent findings showing that encapsulation and incorporation into films/hydrogels protect EGCG, enhancing stability and enabling controlled release [37,38,39,40]. Despite these promising outcomes, non-covalent assemblies may be inadequate for long-term applications requiring thermal, oxidative, or pH stability. Moreover, these studies remain fragmented, addressing individual structural or functional aspects without a broader integrative perspective. In particular, systematic evaluation of the structural, oxidative, and biological implications of alginate–EGCG conjugates remains limited. We have developed various EGCG-conjugated biocompatible polymers such as poly(ethylene glycol), hyaluronic acid, and poly(acrylic acid), functionalizing them for advanced bioapplications [29,33,34,41,42]. Expanding knowledge in this area can support the development of advanced antioxidant materials for safer, more stable integration into food, cosmetic, and biomedical products.

In this study, we developed and characterized alginate–EGCG conjugates with tunable degrees of substitution (DS) by the nucleophilic addition reaction between EGCG quinone and thiolated alginate at a neutral pH in an aqueous medium. We evaluate the resulting conjugate in terms of inhibition of autoxidation, antioxidant capacity, and in vitro safety, varying DS, and compare its performance to free EGCG, alginate, and a non-covalent mixture of EGCG and alginate. We demonstrate that natural polymer conjugation not only protects EGCG from decomposition but also enhances its functional longevity and suitability for its beneficial applications. Furthermore, DS-dependent particle formation via self-assembly was investigated. This work may offer significant knowledge to advance the rational design of natural polymer–polyphenol conjugates for safe and enhanced antioxidant applications across food, health, and consumer products, contributing to the growing field of polymer–polyphenol conjugates as bioactive platforms in oxidative deterioration management.

## 2. Results and Discussion

### 2.1. Synthesis and Characterization of Alginate–EGCG Conjugates

**Figure 1:**
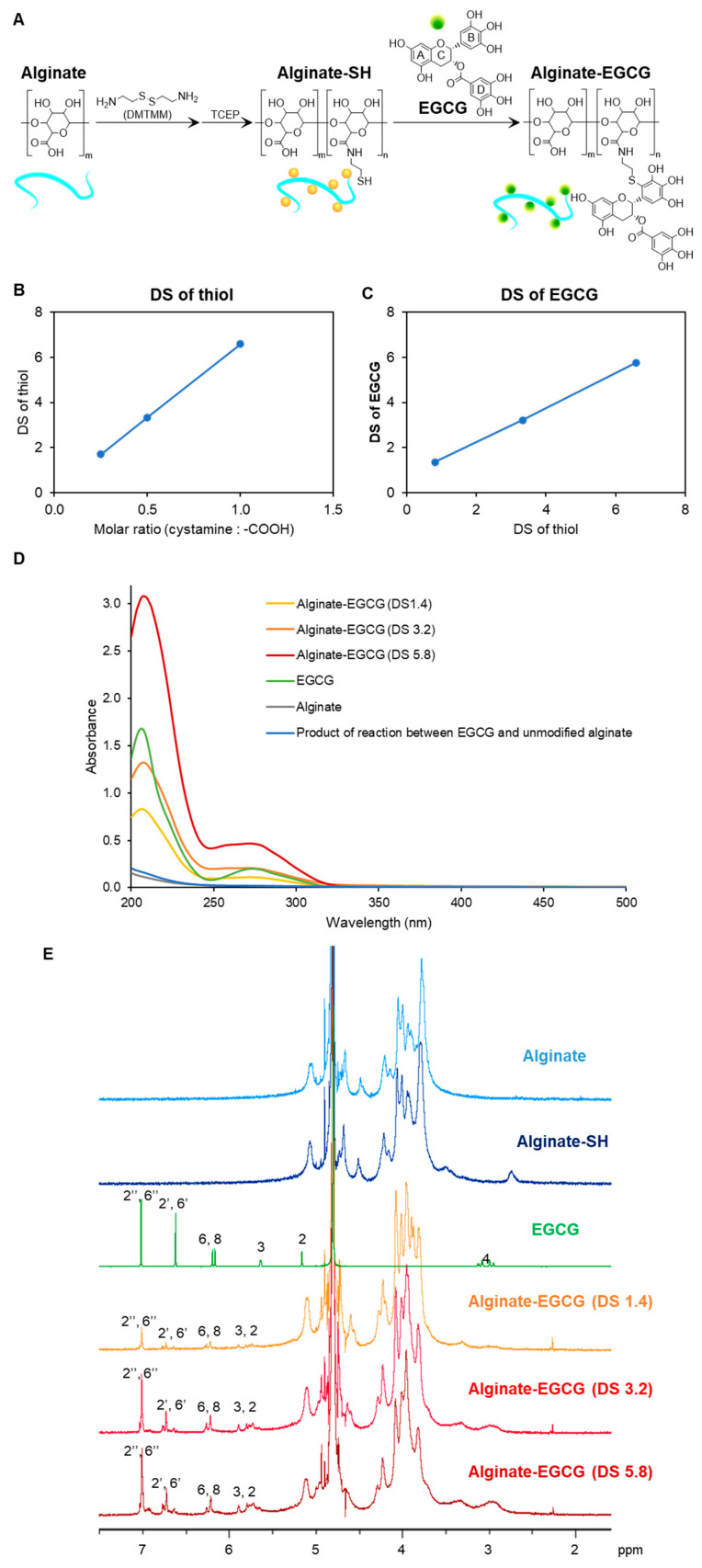
Synthesis of alginate–EGCG conjugate. (**A**) Proposed synthetic scheme. (**B**) DS of thiolated alginate (alginate-SH conjugate) synthesized at different molar ratios of cystamine to carboxyl group. (**C**) Relation between DS of alginate–EGCG conjugates and DS of alginate-SH conjugates. (**D**) UV−visible spectra of alginate–EGCG conjugate (DS = 1.4, 3.2, and 5.8, 0.125 mg/mL), EGCG (8 μg/mL, equivalent to alginate–EGCG with DS = 3.2), sodium alginate (0.125 mg/mL), and a product of the reaction between EGCG and unmodified alginate (0.125 mg/mL), and (**E**) ^1^H NMR spectrum of alginate, alginate-SH conjugate, EGCG, and alginate–EGCG conjugate (DS = 1.4, 3.2, and 5.8) in D_2_ O. mean ± sd (*n* = 3).

Figure 1A illustrates the synthetic strategy for alginate–EGCG conjugates, which involves two sequential reactions designed to enable site-specific conjugation while preserving the structural integrity of EGCG. This method was developed to enhance the oxidative stability and bioactivity of EGCG, a polyphenolic compound derived from green tea renowned for its potent antioxidant and therapeutic properties, but hindered by poor stability under physiological conditions. The two-step process covalently links EGCG to the hydrophilic and biocompatible polysaccharide sodium alginate, offering a stabilized formulation suitable for various biomedical and nutraceutical applications.

In the first step of the synthetic process, cystamine was coupled to carboxyl groups of sodium alginate using 4-(4,6-dimethoxy-1,3,5-triazin-2-yl)-4-methylmorpholinium chloride (DMTMM), a triazine-based coupling agent known to promote efficient amide bond formation in aqueous environments [43]. Sodium alginate, a naturally occurring linear copolymer composed of β-D-mannuronic acid and α-L-guluronic acid, is rich in carboxylic acid functionalities that serve as reactive sites for conjugation. Cystamine, a diamine containing a central disulfide bond, was chosen as the linker due to its functional thiol potential after reduction. The formation of amide bonds between the carboxyl groups of alginate and the amine groups of cystamine yielded a disulfide-bridged intermediate polymer. This intermediate was subsequently reduced using Tris(2-carboxyethyl)phosphine hydrochloride (TCEP), a selective and efficient reducing agent that cleaves disulfide bonds without disrupting other functional groups. The reduction in the disulfide bond in cystamine introduced free thiol groups onto the alginate backbone, resulting in the formation of thiol-functionalized alginate (alginate–SH conjugate). These thiol groups serve as nucleophilic sites for subsequent conjugation reactions with electrophilic compounds. Notably, the DS of thiol groups on alginate-SH conjugates was found to be tunable based on the molar ratio of cystamine to carboxylic acid groups in the feed solution, increasing from 1.7 to 6.6 as the feed ratio increased from 0.3 to 1.0 (Figure 1B). This tunability is critical because it allows precise control over the density of reactive sites on the polymer backbone, which in turn determines the extent of EGCG conjugation and the overall functionality of the final conjugate.

In the second step, EGCG was conjugated to the alginate-SH conjugates at a slightly alkaline pH (7.4), where EGCG is prone to autoxidation, particularly at the pyrogallol moiety located on its B-ring [44]. Under these conditions, EGCG undergoes oxidation to form electrophilic ortho-quinone structures. These quinones are highly reactive and serve as ideal targets for nucleophilic attack by thiol groups. The reaction proceeds via a Michael-type addition mechanism, where the nucleophilic thiol group of alginate–SH conjugates attacks the electrophilic carbon of the quinone, followed by structural rearrangement to form a stable carbon-sulfur (C–S) bond [45]. Importantly, this reaction occurs preferentially at C2′ in the B-ring of EGCG, preserving the polyphenolic structure of EGCG, which is critical for its antioxidant and biological activities. The DS of EGCG on the alginate backbone was found to be linearly correlated with the DS of the thiol-functionalized alginate precursor, as shown in Figure 1C. This result indicates that the extent of EGCG conjugation can be predictably modulated by adjusting the initial thiol content of the alginate–SH conjugates. Such a predictable relationship enhances the reproducibility of the conjugation process and supports the development of tailored antioxidant materials for specific biomedical or food-related applications. All alginate–EGCG conjugates, regardless of their DS values (ranging from 1.4 to 5.8), remained fully soluble in aqueous media, forming optically clear solutions. It suggests that the conjugation did not significantly disrupt the hydrophilicity of the alginate backbone. This high solubility is particularly advantageous for biomedical applications, where stability in aqueous media is essential. Furthermore, the absence of residual free thiol groups in the final product, as confirmed by Ellman’s assay, indicates that the thiol-quinone reaction proceeded to near completion. This minimizes concerns regarding unreacted thiols that could undergo undesirable oxidative side reactions or form disulfide cross-linked networks.

To verify successful conjugation and assess the structural integrity of EGCG post-conjugation, UV-visible spectroscopy was performed. An absorption peak at 274 nm, characteristic of the intact EGCG, was observed for all conjugates (Figure 1D) [41,46]. This finding indicates that the essential flavanol structure of EGCG was retained during the conjugation process. Notably, the broad absorption peak typically associated with EGCG oxidative degradation products, centered around 425 nm [47], was absent in the conjugates. This suggests that the covalent conjugation of EGCG to alginate significantly stabilizes the compound against autoxidation during both synthesis and storage. To rule out the possibility of non-covalent interactions accounting for the observed spectral features, a control reaction was conducted between EGCG and unmodified alginate. This control did not exhibit the 274 nm absorption peak, thereby confirming that the final product did not contain unconjugated EGCG associated with alginate through mere physical interactions. Further structural characterization was carried out using ^1^H NMR spectroscopy (Figure 1E). The ^1^H NMR spectra of alginate–EGCG conjugates revealed preserved proton signals corresponding to the A-ring (H-6, H-8 at 6.2–6.3 ppm), C-ring (H-2, H-3 at 5.6–5.9 ppm), and D-ring (H-2″, H-6″ at 7.0 ppm) of EGCG, indicating that these rings remained chemically unaltered [45]. These moieties are known to play important roles in EGCG’s biological activity, including metal ion chelation and radical scavenging.

Conversely, the proton signals corresponding to the B-ring (H-2′ and H-6′) were reduced to approximately half the intensity of the D-ring signals, suggesting mono-substitution at the B-ring. This is consistent with the site-specific mechanism of thiol addition to the oxidized B-ring of EGCG [34]. This selective reactivity is a major advantage of the thiol-quinone coupling strategy. Many traditional chemical modifications of polyphenols often risk non-specific reactions that impair their bioactivity of polyphenols by disrupting their redox-active functions. In contrast, the strategy demonstrated here ensures conjugation occurs at a defined position, preserving the structural elements essential for antioxidant activity while minimizing potential side reactions. Additionally, the site-specific approach also limits the formation of oligomers or uncontrolled cross-linked networks, which can occur when quinones react with multiple nucleophilic sites, leading to aggregation or poor solubility. The strategy demonstrated here provides high conjugation efficiency, excellent structural preservation, and customizable substitution levels.

### 2.2. Stability Against Autoxidation

**Figure 2:**
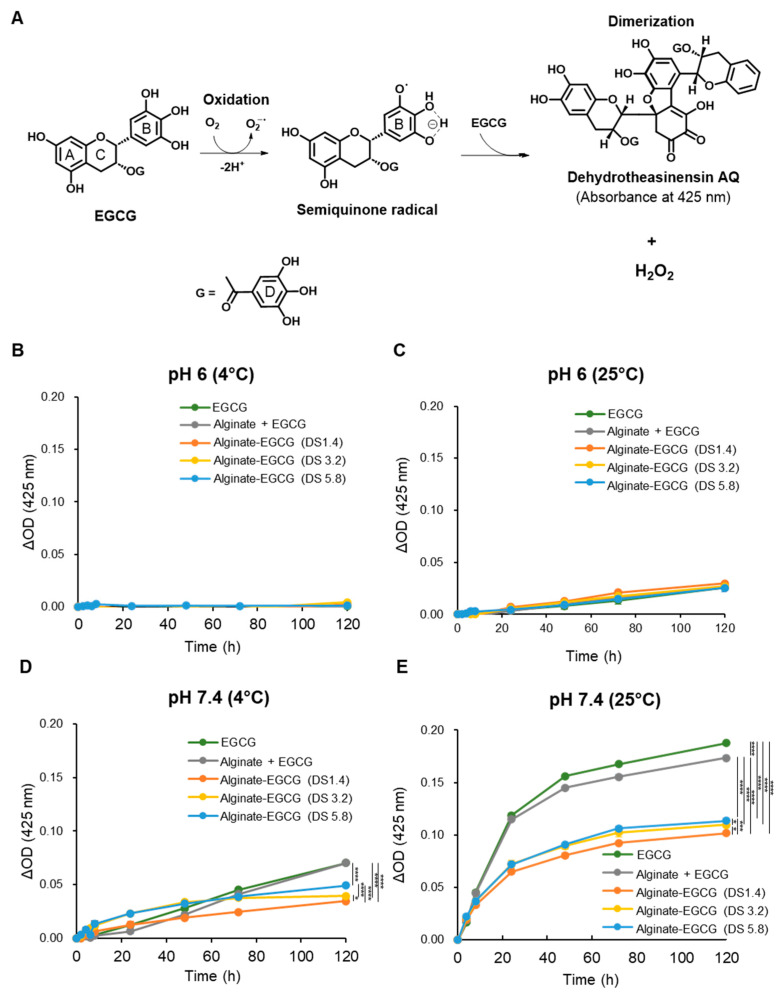
(**A**) Possible mechanism for EGCG autoxidation. (**B**) Delta absorbance for EGCG, a mixture of alginate and EGCG (equivalent concentration to the conjugate with DS = 3.2), and alginate–EGCG conjugates (DS = 1.4, 3.2, and 5.8) at pH 6 under 4 °C and (**C**) 25 °C, (**D**) at pH 7.4 under 4 °C and (**E**) 25 °C, at an equivalent EGCG concentration (100 μM). mean ± sd (*n* = 3). Statistically significant differences in the final values between groups are marked as asterisks; * *p* < 0.05; *** *p* < 0.005; **** *p* < 0.001. Error bars are not displayed because the standard deviations were within the line thickness, indicating negligible variation among replicates.

EGCG possesses potent antioxidant activity but is prone to rapid autoxidation under neutral to alkaline pH and oxygen exposure, generating ROS such as hydrogen peroxide (H_2_O_2_) and semiquinone radicals that diminish its efficacy and safety [14]. In alkaline aqueous environments exposed to atmospheric oxygen, EGCG undergoes a well-characterized autoxidation pathway. Initially, it donates an electron to molecular oxygen, generating superoxide anion radicals (O_2_^•−^), which in turn abstract additional electrons to form semiquinone radicals. These intermediates, particularly reactive at the B-ring of the EGCG molecule, further react with either additional EGCG or oxygen species to form dimers such as dehydrotheasinensin AQ and other oxidized derivatives (Figure 2A). This behavior restricts the formulation and storage of EGCG in pharmaceutical, cosmetic, and food applications. Therefore, enhancing the oxidative stability of EGCG is essential for its broader and feasible application.

To investigate whether alginate conjugation could stabilize EGCG, we evaluated the oxidative decomposition behavior of both free EGCG and alginate–EGCG conjugates under various physicochemical conditions. The hallmark of EGCG autoxidation is a characteristic increase in absorbance at approximately 425 nm, corresponding to the formation of chromophoric oxidation products, such as dehydrotheasinensin AQ [14]. This wavelength thus serves as a convenient optical marker to monitor EGCG decomposition over time. To quantify and compare the decomposition profiles, free EGCG and alginate–EGCG conjugates (equivalent EGCG concentration, 100 μM) were incubated in phosphate-buffer solutions at pH 6.0 and pH 7.4, under two temperature conditions (4 °C and 25 °C) for 5 days while exposed to atmospheric oxygen. This matrix of environmental variables was chosen to simulate both mild and physiologically relevant stress conditions. As anticipated, free EGCG and alginate–EGCG conjugates showed very limited increase in absorbance at 425 nm at pH 6 under 4 °C (Figure 2B), indicating minimal autoxidation under mildly acidic and cool conditions. However, even at this pH, raising the temperature to 25 °C led to a moderate rise in absorbance (Figure 2C), confirming that temperature alone can accelerate EGCG decomposition, likely by enhancing the kinetic energy of oxygen and EGCG molecules. At neutral pH (7.4), where EGCG’s oxidation rate is known to accelerate, substantial increases in 425 nm absorbance were observed, particularly at 25 °C (Figure 2D,E). This underscores the dual role of pH and temperature in modulating the redox stability of EGCG. The oxidative stress under such conditions mimics that of physiological and ambient environments, underlining the importance of stabilization techniques for EGCG-based therapeutic and nutritional products.

In contrast, alginate–EGCG conjugates displayed a marked resistance to autoxidation under all tested conditions. Notably, at pH 7.4 under 25 °C, which was the most oxidative of the studied conditions, the increase in absorbance at 425 nm was substantially suppressed in alginate–EGCG conjugates compared to free EGCG. This protective effect was notably absent in the physical mixture of EGCG with alginate, which exhibited decomposition kinetics almost similar to free EGCG, suggesting that simple mixing does not provide comparable oxidative resistance. The enhanced oxidative stability in the conjugates can be attributed to multiple mechanistic factors. First, covalent conjugation effectively immobilizes EGCG molecules along the alginate polymer chain, reducing their ability to aggregate and undergo radical propagation reactions. This spatial isolation likely decreases intermolecular electron transfer events that are prerequisites for semiquinone and dimer formation. Second, the alginate matrix, being hydrophilic and viscous, may serve as a physical barrier limiting the diffusion of oxygen and ROS into close proximity with EGCG moieties [29,46]. Third, conjugation at the B-ring may alter the electron density of the EGCG molecule in a manner that suppresses its reactivity toward molecular oxygen or nucleophilic agents. This electronic stabilization is particularly valuable in preserving the phenolic hydroxyls essential for EGCG’s bioactivity while minimizing unwanted side reactions.

Interestingly, the DS of EGCG on the alginate backbone influenced oxidative stability. While all conjugates were more stable than free EGCG, those with lower DS values (e.g., DS = 1.4) exhibited superior resistance to autoxidation. In contrast, conjugates with higher DS values (e.g., DS = 5.8) exhibited a higher absorbance at 425 nm under elevated pH and temperature conditions; however, the levels remained substantially lower than those observed for free EGCG. This finding suggests that excessively high local concentrations of EGCG on the polymer backbone may facilitate intramolecular or adjacent intermolecular radical propagation, partially negating the protective effect of conjugation. In other words, the protective effects of conjugation may be compromised at high DS due to overcrowding of EGCG moieties and reduced spatial separation. By comparison, a physical mixture of EGCG and alginate at an equivalent polymer concentration to the conjugate with DS = 3.2 exhibited a similar or minimal reduction in absorbance at 425 nm compared to free EGCG (Figure 2B–E). These findings emphasize the superiority of chemical conjugation over physical mixtures. The advantage of covalent conjugation lies in its ability to tailor spatial presentation, electron distribution, and physical shielding in a single integrated strategy.

Collectively, covalent conjugation of EGCG to alginate significantly enhances its stability against autoxidation by most likely preventing radical propagation, limiting oxygen exposure, and maintaining the structural integrity of the phenolic rings. The enhanced resistance is most pronounced under conditions where free EGCG is highly unstable, such as elevated pH and temperature. This strategy far exceeds the protective capabilities of physical entrapment and offers a rational, tunable approach to improve the functional lifespan of polyphenols.

### 2.3. Hydrogen Peroxide Production

H_2_O_2_ is a key intermediate generated during EGCG autoxidation and serves as a useful marker of oxidative instability [48]. Thus, measuring H_2_O_2_ production offers valuable insight into the oxidative stability of EGCG and the potential for ROS-mediated cytotoxicity in biological applications.

**Figure 3:**
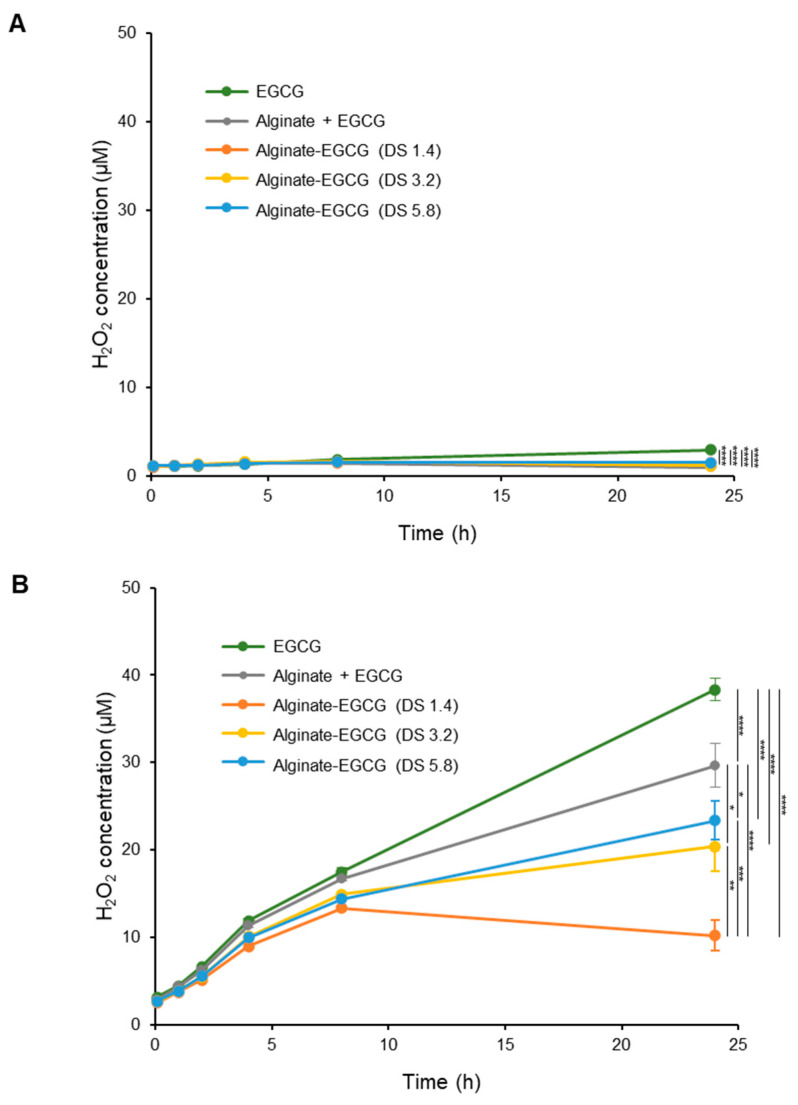
Time-course production of H_2_O_2_ of EGCG, a mixture of alginate and EGCG (equivalent concentration to the conjugate with DS = 3.2), and alginate–EGCG conjugates (DS = 1.4, 3.2, and 5.8) at an equivalent EGCG concentration (100 μM) at (**A**) pH 6 and (**B**) pH 7 under 25 °C. mean ± sd (*n* = 3). Statistically significant differences in the final values between groups are marked as asterisks; * *p* < 0.05; ** *p* < 0.01, *** *p* < 0.005; **** *p* < 0.001.

To investigate the extent to which covalent conjugation of EGCG to alginate mitigates H_2_O_2_ formation, we conducted a comparative analysis between free EGCG and alginate–EGCG conjugates under different pH conditions. Samples containing 100 μM EGCG-equivalent concentrations were incubated in phosphate buffer at pH 6 and pH 7.0 under 25 °C. The time-dependent production of H_2_O_2_ was quantified using a sensitive colorimetric assay. EGCG incubated under a mildly acidic condition (pH 6) yielded only trace amounts of H_2_O_2_, demonstrating that acidic environments slow down the redox cycling of EGCG (Figure 3A). At pH 7, free EGCG rapidly generated H_2_O_2_, consistent with previous reports that alkaline environments accelerate EGCG autoxidation (Figure 3B). In contrast, alginate–EGCG conjugates produced significantly less H_2_O_2_ across the same time frame under both pH conditions, indicating a robust suppression of the oxidative pathway with the protective effects across a physiologically relevant pH range. This implied that conjugation of EGCG to alginate not only suppresses immediate oxidation but also attenuates downstream ROS generation, including harmful species like H_2_O_2_. This suppression was particularly notable in conjugates with lower DS values, such as DS = 1.4, where the production of H_2_O_2_ was minimal. On the other hand, conjugates with higher DS (e.g., DS = 5.8) exhibited a moderate but still significantly reduced H_2_O_2_ output compared to free EGCG. Interestingly, a physical mixture of EGCG (100 μM) and alginate (equivalent concentration to alginate–EGCG conjugate with DS = 3.2) resulted in a modest reduction in H_2_O_2_ levels compared to free EGCG, although its inhibition effect was significantly less compared to the covalent conjugate with DS = 3. This result may be attributed to the increased viscosity and restricted diffusion environment provided by the polymer network, which could impede radical propagation to a limited extent. However, the protective effect was notably less than that observed in covalent conjugates, again underscoring the superior stabilizing power of chemical linkage over physical blending.

**Figure 4:**
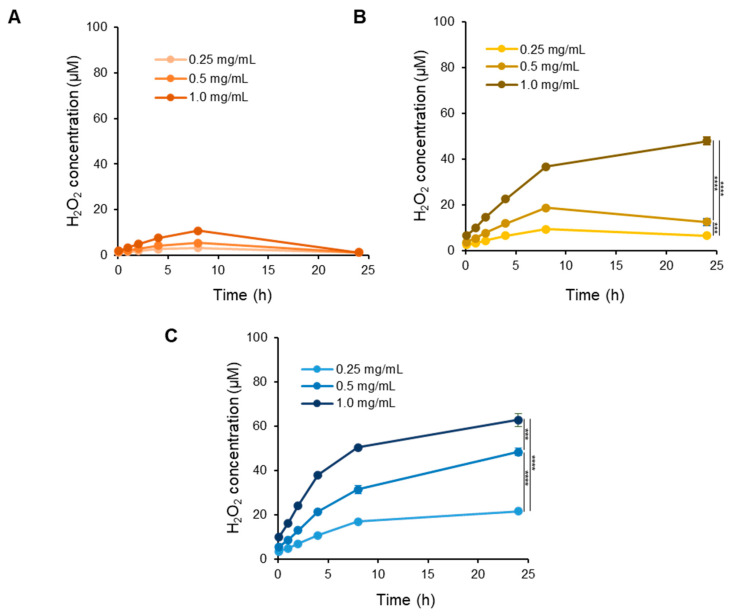
Time-course production of H_2_O_2_ of alginate–EGCG conjugates with (**A**) DS = 1.4, (**B**) DS = 3.2, and (**C**) DS = 5.8, varying concentrations at pH 7 under 25 °C. mean ± sd (*n* = 3). Statistically significant differences in the final values between groups are marked as asterisks; *** *p* < 0.005; **** *p* < 0.001.

Figure 4 presents the concentration- and time-dependent phenomena in H_2_O_2_ production for various DS values. The rate of H_2_O_2_ generation was initially linear for up to 4 h, after which a plateau was reached, implying that the system had reached the equilibrium between H_2_O_2_ production and its decomposition [49]. As EGCG loading increased, a corresponding increase in H_2_O_2_ production was observed. This supports the hypothesis that higher EGCG density on the polymer may increase the risk of redox cycling if spatial separation between moieties is insufficient. It was considered that the reduced proximity of EGCG and a diffusional barrier of alginate would play an important role in decreasing H_2_O_2_ production. In addition, conjugating alginate at the B-ring may create steric hindrance, restricting oxygen accessibility and thus reducing the reactivity of key hydroxyl groups responsible for initiating autoxidation of EGCG, consequently decreasing ROS generation potential.

The biological implications of reducing H_2_O_2_ formation are significant. H_2_O_2_ plays dual roles in cells: it acts as a signaling molecule at low concentrations but becomes cytotoxic at elevated levels, contributing to oxidative damage, mitochondrial dysfunction, DNA damage, and inflammation. In the context of chronic non-communicable diseases (NCDs) such as neurodegeneration, cancer, and cardiovascular disease, the chronic overproduction of H_2_O_2_ is a major pathogenic factor. Therefore, formulations that minimize H_2_O_2_ production while maintaining antioxidant function are of substantial therapeutic interest. The results demonstrate that EGCG conjugation to alginate not only stabilizes EGCG structurally but also functionally by limiting ROS generation. This property is especially desirable in biomedical formulations intended for long-term use or in oxidative stress-sensitive systems. Furthermore, the ability to fine-tune H_2_O_2_ production through adjustment of DS introduces a new dimension in the design of antioxidant systems—one that combines efficacy with safety.

### 2.4. Cytotoxicity

ROS such as H_2_O_2_ and semiquinone radicals are key mediators of cellular injury and are implicated in cancer, neurodegeneration, cardiovascular, and inflammatory disorders [50]. At neutral to alkaline pH, EGCG is prone to autoxidation, forming semiquinone intermediates and reactive byproducts, including H_2_O_2_. These reactive species can disrupt cellular homeostasis, damage DNA, lipids, and proteins, and trigger pro-inflammatory and apoptotic pathways. This oxidative liability significantly limits the clinical translation of EGCG, as it introduces potential cytotoxic effects in normal tissues.

**Figure 5:**
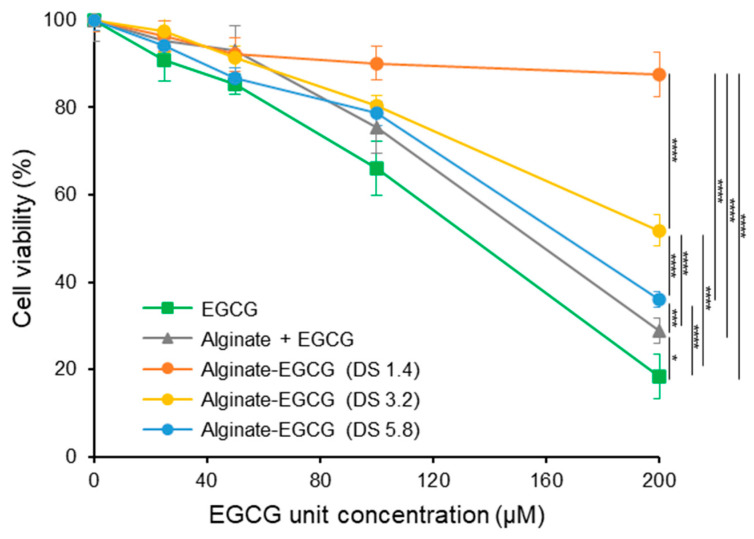
Cell viability of human primary renal proximal tubule epithelial cells (RPTEC) treated by EGCG, a mixture of alginate and EGCG (equivalent concentration to the conjugate with DS = 3.2), and alginate–EGCG conjugates (DS = 1.4, 3.2, and 5.8) as a function of concentration for 24 h. mean ± sd (*n* = 5). Statistically significant differences in the final values between groups are marked as asterisks; * *p* < 0.05; *** *p* < 0.005; **** *p* < 0.001.

To examine whether conjugation of EGCG to alginate could mitigate its intrinsic cytotoxicity, we conducted in vitro cytotoxicity assays using human renal proximal tubule epithelial cells (RPTECs). These cells are sensitive to oxidative damage and serve as a representative model for evaluating systemic toxicity of bioactive compounds. The cells were exposed to increasing concentrations of free EGCG and alginate–EGCG conjugates with varying DS. As expected, free EGCG demonstrated a dose-dependent reduction in cell viability. At concentrations below 100 μM, EGCG exhibited mild cytotoxicity; however, at 200 μM, a substantial decline in viability was observed, dropping to 18.4% (Figure 5). This sharp decrease reflects the cytotoxic effects of ROS accumulation from EGCG autoxidation and aligns with prior studies reporting EGCG-induced ROS-mediated cytotoxicity in various cell types [50]. In stark contrast, the alginate–EGCG conjugates displayed markedly improved cytocompatibility. Across all tested concentrations, conjugates with low DS values (e.g., DS = 1.4) maintained high cell viability, even at 200 μM EGCG-equivalent concentration. Specifically, DS = 1.4 yielded 87.6% viability, representing an almost five-fold improvement compared to free EGCG. This dramatic enhancement suggests that conjugation substantially suppresses ROS formation and subsequent cellular stress. The polymeric scaffold likely limits the availability of redox-active centers, disperses EGCG moieties spatially, and provides steric protection, thereby mitigating local oxidative bursts. Interestingly, conjugates with higher DS values, such as DS = 3.2 and DS = 5.8, still exhibited reduced cytotoxicity relative to free EGCG but to a lesser extent than low DS conjugates. At 200 μM, DS = 3.2 yielded a viability of 51.8%, while DS = 5.8 yielded a viability of 36.1%. This pattern mirrors trends observed in H_2_O_2_ generation and supports the hypothesis that excessive loading of EGCG increases the risk of intramolecular or local intermolecular radical propagation. High DS may result in insufficient spatial separation between EGCG moieties, thereby promoting redox cycling even within a stabilizing matrix. Thus, optimal DS must balance antioxidant potency and cytoprotective efficacy.

To further validate the importance of covalent bonding, we examined a physical mixture of EGCG and alginate at equivalent concentrations to the conjugate with DS = 3. This mixture resulted in modest improvement in cell viability (28.8% at 200 μM of EGCG), better than free EGCG (18.4%) but significantly inferior to covalently conjugated forms (51.8%). The limited effect of the physical mixture may stem from the ability of alginate to modestly reduce ROS diffusion or aggregate formation through increased viscosity. However, without chemical linkage, EGCG remains vulnerable to autoxidation, and the local concentration of active ROS likely remains high. The results showed good agreement in line with autoxidation and H_2_O_2_ production of EGCG observed earlier. Accordingly, the cytoprotective effect of alginate–EGCG conjugates can be attributed to suppression of EGCG autoxidation and its toxic byproduct generation, demonstrating that the conjugate can provide a more stable, bioavailable, and biocompatible form of EGCG suitable for sensitive biological environments. The implications of these findings are profound, particularly in applications targeting chronic diseases or requiring prolonged exposure, such as oral supplements, injectable therapeutics, or tissue scaffolds. For example, in chronic kidney disease, oxidative stress plays a major pathogenic role, and RPTECs are among the primary cell types affected. Alginate–EGCG conjugates may offer a way to facilitate sustained antioxidant therapy without exacerbating oxidative damage or compromising renal function. This multifaceted protective mechanism also demonstrates the value of polymer conjugation strategies in preserving the beneficial properties of phenolic antioxidants while suppressing their potential toxicity.

### 2.5. Superoxide Anion Radical Scavenging Activity

The ability of EGCG to act as a potent antioxidant is strongly associated with its capacity to neutralize ROS, particularly through scavenging O_2_^•−^, one of the primary free radicals generated during oxidative stress. The antioxidant activity of EGCG is primarily attributed to its unique polyphenolic structure, which contains multiple hydroxyl groups on its B and D rings. These hydroxyl moieties can readily donate hydrogen atoms or electrons to unstable radicals, thereby terminating radical chain reactions and preventing oxidative damage to vital biomolecules such as proteins, lipids, and nucleic acids [51,52]. Beyond direct radical scavenging, EGCG also plays an indirect antioxidant role by chelating redox-active transition metal ions like Fe^2+^ and Cu^2+^. These metals catalyze Fenton-type reactions, which convert H_2_O_2_ into highly reactive hydroxyl radicals (•OH) [50]. By binding to these ions, EGCG can inhibit these harmful reactions and further mitigate oxidative damage. Furthermore, EGCG has been reported to inhibit the activity of ROS-generating enzymes such as xanthine oxidase (XO). XO catalyzes the oxidation of hypoxanthine and xanthine to uric acid while simultaneously generating O_2_^•−^. EGCG’s ability to competitively inhibit XO further enhances its antioxidant potential [50].

**Figure 6:**
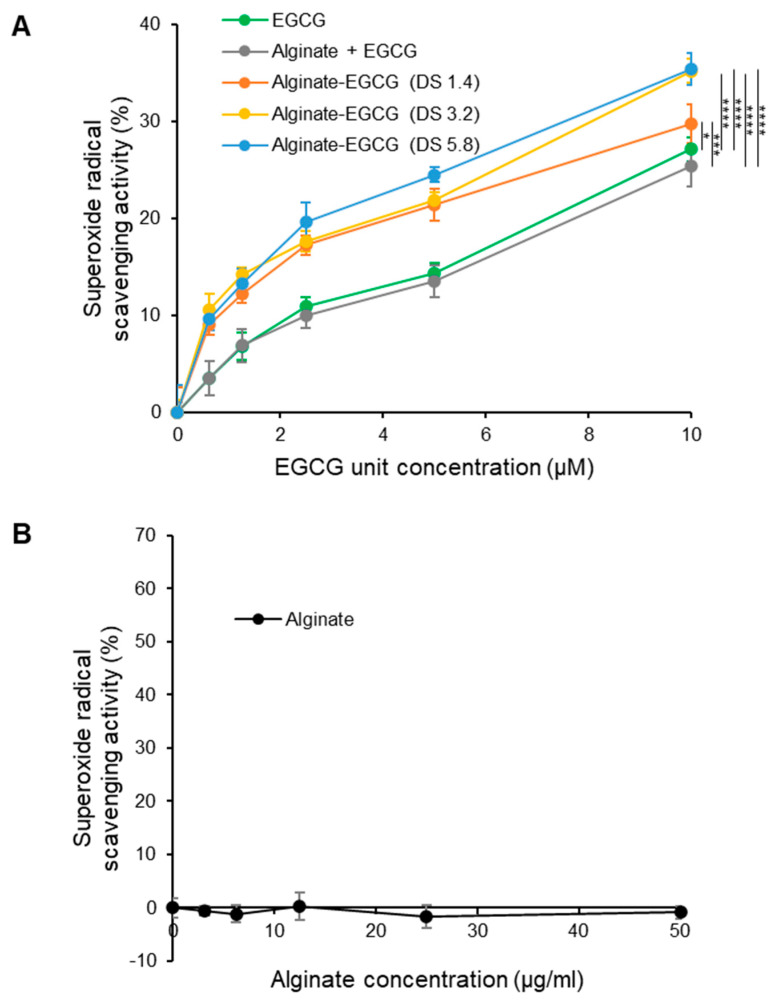
Superoxide anion radical scavenging activities of (**A**) EGCG, a mixture of alginate and EGCG (equivalent concentration to the conjugate with DS = 3.2), and alginate–EGCG conjugates (DS = 1.4, 3.2, and 5.8) as a function of concentration, and (**B**) alginate (equivalent concentration range tested for compared samples) at pH 7 and 37 °C. mean ± sd (*n* = 3). Statistically significant differences in the final values between groups are marked as asterisks; * *p* < 0.05; *** *p* < 0.005; **** *p* < 0.001.

To investigate the influence of this covalent conjugation of EGCG to alginate on its antioxidant capacity, we assessed radical scavenging activity, particularly its ability to scavenge O_2_^•−^, excessive production of which can induce oxidative stress, leading to biomolecular damage and contributing to various pathological conditions. This was evaluated using a standard in vitro xanthine/XO assay system, which generates O_2_^•−^ in a controlled and quantifiable manner. Both free EGCG and alginate–EGCG conjugates were tested across a range of concentrations, all normalized to the equivalent EGCG content to allow fair comparison. As illustrated in Figure 6A, all samples demonstrated concentration-dependent scavenging of O_2_^•−^, confirming the inherent antioxidant activity of EGCG. Remarkably, the alginate–EGCG conjugates consistently outperformed free EGCG in a whole range of concentrations tested. This enhancement in activity would be attributed to covalent attachment to alginate that stabilizes the redox-active hydroxyl groups of EGCG, preventing their premature autoxidation and preserving their functionality over time. The DS of alginate–EGCG conjugates also influenced antioxidant activity. Higher DS values, corresponding to greater EGCG content per alginate chain, generally exhibited stronger radical scavenging activity. This can be explained by the increased availability of active phenolic hydroxyl groups per polymer unit. Importantly, even at the highest tested DS (DS = 5.8), no evidence of aggregation-induced self-quenching or steric hindrance due to overcrowding of EGCG molecules was observed, indicating that the conjugate architecture remains favorable for antioxidant function within this range. The physical mixture of free EGCG and alginate displayed a scavenging profile nearly identical to that of free EGCG alone, suggesting that physical entrapment within the alginate matrix provides no functional advantage in radical quenching. This result highlights the necessity of chemical conjugation for achieving the enhancement of antioxidant properties. This superior antioxidant activity of the covalently conjugated EGCG to the hydrophilic polymer over the non-conjugated mixture system was in good agreement with previous reports [53].

Unmodified alginate alone was also tested in a range of equivalent concentrations to the compared samples, since unmodified polymers often showed modest ROS scavenging activities through physically restricted movement of ROS due to the viscous polymer meshwork. Alginate exhibited negligible scavenging activity across the entire concentration range (Figure 6B). From a material design standpoint, these findings demonstrate the feasibility of fine-tuning antioxidant performance by precisely adjusting conjugation parameters, particularly DS. The results reveal an inherent stability-biofunction trade-off: while increasing EGCG loading enhances antioxidant capacity, it may also elevate redox reactivity if the spatial distribution of EGCG moieties is not optimized. This interplay between stability and bioactivity can be strategically exploited to develop formulations with tailored antioxidant functions and defined shelf-life requirements.

From a formulation perspective, optimized alginate–EGCG conjugates could be applied in antioxidant systems where stability is critical, such as dietary supplements, pharmaceutical excipients, or bioactive coatings. Low-DS conjugates may be preferable for sustained, low-level antioxidant delivery in applications such as cosmetics or functional foods, whereas high-DS conjugates may be suited for short-term, high-potency interventions in therapeutic formulations targeting acute oxidative injury. The tunable relationship between antioxidant content and ROS-suppression capacity offers additional flexibility for designing systems with precise redox profiles.

The observed enhancement in O_2_^•−^ scavenging activity is particularly relevant in the context of chronic NCDs, where excessive superoxide generation contributes to the pathogenesis of conditions such as atherosclerosis, diabetic nephropathy, and neuroinflammation. By efficiently scavenging superoxide radicals and maintaining this activity through covalent stabilization, alginate–EGCG conjugates represent a promising strategy to mitigate ROS-mediated damage and inflammation. Their combination of superior antioxidant performance, biocompatibility, and solubility supports their potential for integration into oral, topical, or injectable delivery systems.

### 2.6. Particle Formation by Self-Assembly

EGCG possesses amphiphilic properties, with aromatic rings driving hydrophobic interactions (π–π stacking, hydrogen bonding) and phenolic hydroxyls conferring hydrophilicity. When conjugated to the anionic polysaccharide alginate, these features promote spontaneous self-assembly into core–shell nanoparticles: EGCG-rich hydrophobic moieties aggregate to form the core, while alginate chains extend into the aqueous medium, providing steric and electrostatic stabilization. This architecture enhances colloidal stability and offers advantages for controlled release and targeted delivery.

**Figure 7:**
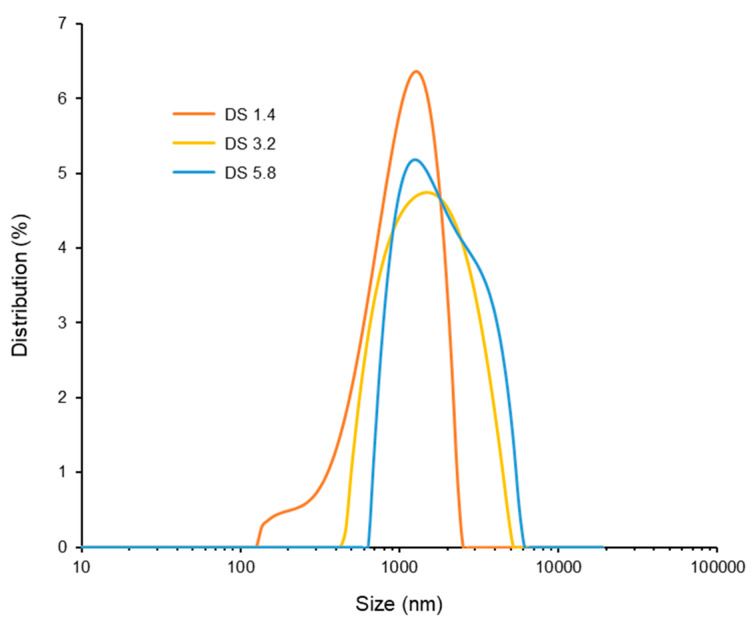
Size distribution of alginate–EGCG conjugates with DS = 1.4, 3.2, and 5.8 at 10 mg/mL. mean ± sd (*n* = 3).

To investigate the self-assembly behavior and particle formation of alginate–EGCG conjugates, dynamic light scattering (DLS) analysis was performed in aqueous solutions under ambient conditions. The DS of EGCG on the alginate backbone was varied to examine its influence on particle formation. As shown in Figure 7, increasing the DS from 1.4 to 5.8 led to a corresponding increase in hydrodynamic particle diameter. Specifically, the average particle sizes followed the trend: DS 1.4 (1092.3 ± 60.0 nm) < DS 3.2 (1384.4 ± 37.8 nm) < DS 5.8 (2130.0 ± 485.2 nm), suggesting that a higher EGCG content promotes stronger hydrophobic interactions and leads to the formation of larger assemblies. However, the size distribution was not uniform across all samples. Interestingly, among the tested DS values, alginate–EGCG with DS = 3.2 exhibited the narrowest and most unimodal size distribution (PDI = 0.204), indicating the formation of a more homogeneous particle population. This suggests that DS = 3.2 represents an optimal balance between the hydrophobic driving force of EGCG and the hydrophilic stabilization by alginate, likely yielding stable and well-defined particles. In contrast, conjugates with a lower DS (e.g., DS = 1.4) formed smaller particles, but the distribution was broader (PDI = 0.269). The limited EGCG content may not provide sufficient hydrophobic attraction to drive compact core formation, resulting in less organized structures or loosely packed aggregates. On the other hand, at higher DS (e.g., DS = 5.8), although the particles were larger, their size distribution was wider (PDI = 0.250), indicating increased heterogeneity. This may be due to excessive hydrophobic clustering leading to over-aggregation, including interparticle interactions. These observations emphasize the importance of DS optimization in particle engineering. Tuning the EGCG content along the alginate backbone directly influences the balance between affinitive and repulsive forces governing particle formation. A critical EGCG density is necessary to initiate self-assembly via hydrophobic interactions, but beyond a certain threshold, steric and electrostatic stabilization provided by alginate may be insufficient to prevent aggregation, leading to size heterogeneity and decreased colloidal stability.

The particle formation mechanism in alginate–EGCG conjugates mirrors that of amphiphilic block copolymers, where the hydrophobic and hydrophilic segments segregate to form core–shell micelles. However, the use of natural materials such as EGCG and alginate introduces biocompatibility and inherent bioactivity, which are highly advantageous for applications in drug delivery, nutraceuticals, and food stabilization. The spontaneous assembly in aqueous conditions without the need for organic solvents or surfactants further enhances the environmental and biological safety of the system, compared to amphiphilic block copolymers. Beyond structural advantages, the particle format offers functional benefits.

Collectively, these results demonstrate that the EGCG-to-alginate ratio, as reflected in DS, is a key determinant of particle characteristics. The narrow size distribution observed at DS = 3.2 is particularly valuable for bioactivity-related applications, where uniform particle size can positively influence its performance and safety. Therefore, optimizing the DS of EGCG on alginate provides a powerful strategy for engineering well-defined, self-assembled particles for their functional applications.

## 3. Materials and Methods

### 3.1. Materials

Sodium alginate (Sigma A1112, unknown molecular weight, viscosity: 4–12 cP (1% in H_2_O at 25 °C), monosaccharide repeating unit = 216.12 Da) was purchased from Sigma-Aldrich. 4-(4,6-Dimethoxy-1,3,5-triazin-2-yl)-4-methylmorpholinium chloride (DMTMM, 276.72 Da), cystamine dihydrochloride (225.2 Da), and tris(2-carboxyethyl)phosphine hydrochloride (TCEP, 286.64 Da) were purchased from Tokyo Chemical Industry Co., Ltd. (Tokyo, Japan). EGCG was purchased from Euro Chem-Pharam Sdn Bhd (Shah Alam, Malaysia).

### 3.2. Synthesis and Characterization of Thiolated Alginate

Thiolated alginate derivatives (alginate–SH conjugates) were synthesized by modifying carboxyl groups in the A backbone with thiol groups. Typically, 1 g of sodium alginate (4.63 mmol −COOH) was dissolved in 100 mL of 10 mM phosphate-buffered saline (PBS, pH 7.4). To this solution, equimolar amounts of DMTMM and cystamine dihydrochloride were added to initiate the conjugation process. The reaction mixture was stirred for 24 h at 25 °C. Next, TCEP solution (pH 7) was dropwise added, and the reaction mixture was stirred for an additional 1 h. The resulting solution was transferred to dialysis tubes with a molecular weight cutoff of 3500 Da. The tubes were dialyzed against 0.1 M NaCl solution for 2 days, and deionized water for 2 days in the atmosphere. The purified solution was lyophilized to obtain alginate-SH conjugates. The degree of substitution (DS), defined as the number of substituents per 100 carboxylic groups in alginate, was determined by Ellman’s assay. The resulting alginate–SH conjugates were analyzed by ^1^H NMR (AVANCE III 400 MHz, Bruker Biospin Inc., Rheinstetten, Germany) in D_2_O.

### 3.3. Synthesis and Characterization of Alginate–EGCG Conjugates

Alginate–EGCG conjugates were synthesized by conjugating EGCG to alginate–SH conjugates. In all cases, 0.5 g of alginate–SH conjugates were dissolved in 70 mL of PBS under a nitrogen atmosphere. This solution was added dropwise to 30 mL of PBS containing an excess of EGCG (alginate/EGCG = 1:2.3) with continuous stirring. The pH of the mixture was adjusted to 7.4 by dropwise addition of 1 M NaOH. After reaction for 3.5 h at 25 °C, the pH of the mixture was adjusted to the resultant solution was transferred to dialysis tubes with a molecular weight cutoff of 3500 Da. The tubes were dialyzed against deionized water for 3 days. The purified solution was lyophilized to obtain alginate–EGCG conjugates. The conjugates were analyzed by UV−visible spectra (Jasco V770 spectrophotometer, Jasco Corporation, Tokyo, Japan) and ^1^H NMR spectroscopy. The DS was determined by UV absorbance derived from EGCG at 274 nm. Until the evaluations, the alginate–EGCG conjugates were kept in lyophilized form at −20 °C, and their stability was confirmed by no change in the UV-visible spectra for over one year.

### 3.4. Evaluation of Oxidative Stability

EGCG and alginate–EGCG conjugates with different DS at an equivalent EGCG unit concentration (100 µM) were prepared in 10 mM phosphate solution and incubated at 4 and 25 °C in a dark place. UV-visible spectra of samples were measured at designated time points for 5 days. The formation of EGCG oxidation product (dehydrotheasinensin AQ) was monitored by measuring its absorbance at 425 nm at each time point [47].

### 3.5. Evaluation of H_2_O_2_ Production

The H_2_O_2_ production of samples was determined by using a Pierce Quantitative Peroxide Assay Kit (Thermo Fisher Scientific, Waltham, MA, USA). Various samples were dissolved in phosphate-buffer solution and incubated at 25 °C. After 24 h, sample solutions (20 μL) were mixed with the kit working reagent (200 μL) in 96-well flat-bottomed microassay plates. After 15 min of incubation at 25 °C in the dark, the absorbance at 595 nm was measured using a microplate reader.

### 3.6. Evaluation of Superoxide Anion. Radical Scavenging Activity

Superoxide anion radical (O_2_^•−^) was generated using the xanthine/XO reaction and measured by the nitroblue tetrazolium (NBT) reduction method. Different samples were mixed in a 0.1 M phosphate-buffer solution (pH 7.0) containing XO (0.4 units/mL) and NBT (0.1 mM) in 96-well microplates. The measurement was started with the addition of xanthine (1 mM). After incubation for 10 min at 37 °C, the production of superoxide radical was measured spectrophotometrically at 560 nm using a microplate reader (Tecan Group Ltd., Männedorf, Switzerland). Superoxide scavenging activity was calculated according to the following equation:

*Superoxide anion radical scavenging activity* (%) = (Acontrol − Asample)/Acontrol × 100

where Asample and Acontrol represent the absorbance obtained from reactions with and without a test sample, respectively.

### 3.7. Evaluation of Cytotoxicity

Cell Culture: Primary human renal proximal tubule epithelial cells (RPTECs) were obtained from ATCC (Manassas, VA, USA), and cultured in renal epithelial cell basal medium (ATCC^®^ PCS-400–030™) supplemented with renal epithelial cell growth kit (ATCC^®^ PCS-400–040™).

Cytotoxicity test: RPTECs were seeded (1 × 10^4^ cells/well) in quadruplicate in 96-well microplates and allowed to adhere for 1 day. The culture media were then replaced by media containing the following samples: EGCG, alginate–EGCG conjugates, alginate, or the physical mixture of alginate and EGCG (25–200 µM of EGCG moiety). The concentrations of alginate correspond to the amount of alginate in alginate–EGCG conjugate (DS = 3.2). The cells were incubated at 37 °C in 5% CO. After 24 h, cell viability was measured by using AlamarBlue reagent (Life Technologies, Carlsbad, CA, USA) according to the manufacturer’s protocol. Briefly, the medium was replaced by media containing 10% AlamarBlue reagent. After incubation for 4 h at 37 °C, cell viability was determined by monitoring the fluorescence intensity (λex = 549 nm and λem = 587 nm) using a microplate reader (Tecan Group Ltd., Switzerland). Results were expressed as a percentage of viable cells relative to untreated cells.

### 3.8. Analysis of Self-Assembly

The self-assembly of alginate–EGCG conjugates was evaluated by the dynamic light scattering (DLS) technique. Test solutions were prepared by dissolving alginate–EGCG conjugates in deionized water at 5 mg/mL and equilibrated at 25 °C for 2 min before measurement. The particle size distributions of alginate–EGCG conjugates were characterized using a Litesizer 500 instrument (Anton Paar, Graz, Austria) at 25 °C.

### 3.9. Statistical Analysis

All data were represented as mean ± standard deviation (SD). The statistical significance of differences between mean values was determined by Student’s *t*-test. Multiple comparisons were evaluated by one-way analysis of variance (ANOVA) using OriginPro 2025. A *p*-value of < 0.05 was considered to be statistically significant.

## 4. Conclusions

This study successfully demonstrated the synthesis and functional evaluation of a covalently conjugated alginate and EGCG system that enhances the stability and antioxidant functionality of EGCG. By employing a thiol-quinone coupling strategy at physiological pH, EGCG was selectively conjugated to alginate without compromising its redox-active flavanic structure. The conjugates effectively inhibited EGCG autoxidation, reduced the formation of cytotoxic H_2_O_2_, and exhibited enhanced cytocompatibility, especially at lower degrees of substitution. Importantly, the conjugates retained or even enhanced superoxide scavenging activity and spontaneously formed self-assembled structures in aqueous media. These results highlight the advantages of covalent conjugation over physical mixtures, emphasizing its potential to extend the shelf-life, safety, and therapeutic efficacy of polyphenols. This work provides valuable insights for the rational design of natural polymer and plant-derived polyphenol conjugates as robust antioxidant systems in bioactivity-related and industrial applications. Future work should include testing in real formulations (e.g., pharmaceutical, nutraceutical, or food systems) and expanding biological assays to diverse cell types relevant to oxidative stress and chronic diseases in order to better assess translational potential.

## Data Availability

The data that support the findings of this study are contained within the article.
